# Association between unresolved drug-related problems and 6-month hospital readmissions following pharmacist-led medication reviews: a retrospective observational study

**DOI:** 10.1007/s11096-026-02139-7

**Published:** 2026-04-17

**Authors:** Kaja Zorjan, Barbara Tašker, Astrid Marovič, Maja Petre

**Affiliations:** Central Pharmacy, University Medical Centre Maribor, Maribor, Slovenia

**Keywords:** Clinical pharmacy, Clinical pharmacist intervention, Drug-related problem, Hospital readmission, Medication review

## Abstract

**Introduction:**

Unresolved drug-related problems may cause preventable medication-related harm and contribute to hospital readmissions. Comprehensive medication reviews conducted by clinical pharmacists are used to identify and address drug-related problems in hospital settings. However, the clinical relevance of drug-related problems that remain unresolved after identification, particularly in relation to hospital readmissions, remains unexplored.

**Aim:**

To examine the association between unresolved drug-related problems and 6-month all-cause hospital readmission, and to describe drug-related problem identification, intervention acceptance, and resolution during pharmacist-led comprehensive medication reviews.

**Method:**

A retrospective observational study was conducted in a tertiary hospital (2019–2022). Inpatients and outpatients referred by doctors for a pharmacist-led comprehensive medication review with ≥ 1 identified drug-related problem were included. The primary outcome was all-cause hospital readmission within 6 months following the medication review. Additional process measures included doctors’ acceptance of pharmacists’ recommendations, drug-related problem resolution status assessed based on follow-up documentation at 6 months, and the number of additional drug-related problems identified by pharmacists beyond those detected by doctors.

**Results:**

A total of 177 patients undergoing 185 comprehensive medication reviews were included, in whom 505 drug-related problems were identified (mean 2.7 per review, standard deviation 2.0). Clinical pharmacists identified 73.9% of all drug-related problems and proposed interventions for 88.9% of them. Overall, 70.2% of pharmacists’ recommendations were accepted by doctors. In a patient-level multivariable analysis adjusting for age, sex, comorbidities, medication count, total drug-related problem count, and the number of comprehensive medication reviews, the presence of any unresolved drug-related problems (OR = 4.02, 95% CI: 1.12–14.39, *p* = 0.033) and the number of unresolved drug-related problems (OR = 1.74, 95% CI: 1.10–2.74, *p* = 0.017) were independently associated with higher readmission risk, as was comorbidity count (OR = 1.47, 95% CI: 1.01–2.15, *p* = 0.046).

**Conclusion:**

In this retrospective study, the presence of unresolved drug-related problems following pharmacist-led comprehensive medication reviews was independently associated with four-fold higher odds of hospital readmission within 6 months. These findings highlight unresolved drug-related problems as a strong, independent marker of increased readmission risk. Prospective studies are needed to determine whether interventions that successfully resolve DRPs can causally reduce readmissions.

## Impact statements

- The presence of unresolved drug-related problems was independently associated with approximately four times higher odds of hospital readmission within 6 months in patient-level multivariable analysis, suggesting that unresolved medication issues may represent a marker of increased clinical risk.
- Pharmacist-led medication reviews uncovered numerous previously unrecognized DRPs, underscoring the added value of pharmacists within multidisciplinary care teams.
- Approximately 70% of pharmacists’ recommendations were accepted and were associated with higher DRP resolution rates and lower observed readmission rates.
- This study is among the first to observe an association between unresolved drug-related problems and higher hospital readmission rates, pointing to a potential focus for enhanced medication management.

## Introduction

A drug-related problem (DRP) is an event or circumstance involving drug therapy that actually or potentially interferes with desired health outcomes [1]. DRPs are recognized as contributors to preventable patient harm and adverse clinical outcomes [2]. In hospitals, many readmissions are drug-related and potentially preventable [3]. While medication-related hospital admissions have been widely studied, less attention has been paid to medication-related hospital readmissions [4]. Observational and meta-analytic data suggest many adverse drug events may be prevented [5, 6]. Medication safety has been identified as an international priority by the World Health Organization [7]. Medication errors remain common and are associated with morbidity and mortality. They have been estimated to contribute to mortality in the USA, accounting for over 250,000 deaths annually [8]. In England, an estimated 237 million medication errors occur each year [9]. Both medical doctors (MD) and clinical pharmacists (CP) are trained to identify and resolve DRPs. A fundamental element of pharmacist medication management is medication review, ranging from simple medication list checks to comprehensive medication reviews (CMRs). CMRs aim to optimize prescribing by evaluating therapeutic effectiveness and potential harm in relation to the individual patient and their conditions [10], while also considering medication adherence, potential interactions, required monitoring and patient preferences. Pharmacist interventions in hospitals are well established, with physician acceptance rates between 70 and 90% [11–17], with some studies reporting even higher uptake (> 90%) [18]. A systematic review reported a broader range of 39–100% [19]. Earlier meta-analyses found no clear benefit of pharmacist-led CMRs on readmission outcomes [20]. More recent evidence shows modest reductions in hospital readmissions, ~ 7% relative to standard care in a 2023 Cochrane meta-analysis (RR = 0.93) [21] and 8% in a 2025 meta-analysis [22], with no significant effect on mortality.

CPs provide recommendations to address identified DRPs, which MDs may accept fully, partially, or not at all. Unresolved DRPs remain a major clinical concern because they indicate ongoing medication-related issues that may compromise treatment quality [23]. Evidence indicates that a proportion of DRPs may remain unaddressed in routine care [23–25]. While pharmacist-led CMRs are widely used to identify DRPs, the clinical relevance of unresolved DRPs has been insufficiently explored. It remains unclear to what extent unresolved DRPs following pharmacist-led medication reviews are associated with subsequent hospital readmissions. Unlike previous studies primarily evaluating the effectiveness of medication reviews, this study focuses on the clinical relevance of DRPs that remain unresolved after identification. Assessing this association may help prioritize medication-related risks in routine care.

### Aim

To examine the association between unresolved drug-related problems and 6-month all-cause hospital readmission, and to describe drug-related problem identification, intervention acceptance, and resolution during pharmacist-led comprehensive medication reviews.

## Method

### Study design and population

A retrospective observational study (2019–2022) was conducted at the University Medical Centre Maribor, Slovenia, a 1,316-bed tertiary public hospital. The study included inpatients and outpatients referred by an MD to a CP for a CMR, who had ≥ 1 DRP identified by either the CP or the referring MD.

### Data collection and classification

Data were collected retrospectively from the hospital’s electronic medical records. Variables included demographics (age, sex, care setting), clinical data (number of medications, selected comorbidities), and detailed DRP information. We recorded 6 common chronic comorbidities (hypertension, diabetes, heart failure, atrial fibrillation, chronic kidney disease and major depressive disorder). DRPs were identified and documented as part of routine clinical care and pharmacist-led CMRs. DRPs were classified according to the Pharmaceutical Care Network Europe (PCNE) classification (version 9.1). Each DRP was classified as either potential (no harm yet) or clinically expressed (with observable consequences). For each DRP, the problem category, cause, CP-proposed interventions, MD’s acceptance (defined as full or partial implementation within 2 months following the completion of the CMR), and resolution status after 6 months were recorded. Resolution was assessed independently of physician acceptance. A DRP was classified as resolved when follow-up documentation indicated that the underlying problem was no longer present at the 6-month assessment (e.g., the inappropriate drug was discontinued and no related problem persisted). Partial resolution was recorded when documentation indicated improvement in a DRP, but the problem was not fully eliminated (e.g., dose optimization with partial symptom/laboratory improvement, but further action was still required), including cases where one component of a multi-faceted DRP was addressed while another component remained. A DRP was classified as unresolved when documentation indicated that the problem remained ongoing at the 6-month follow-up, including situations where a recommended change was not implemented (e.g., not accepted or not acted upon) and no alternative action addressing the DRP was documented, or where implementation was unsuccessful (e.g., non-adherence) and the DRP persisted. The identifier (CP or MD) of each DRP was also documented.

### Outcomes and DRP inclusion criteria

The primary outcome was all-cause hospital readmission within 6 months following the CMR, defined as any inpatient admission to the same tertiary institution for any reason during that period. Hospital readmissions were identified using the hospital information system and were limited to the study centre. For patients who underwent more than one CMR, readmission was linked to the most recent CMR. In addition to the primary outcome, three DRP-related analyses were made: (1) MD’s acceptance of CP-proposed interventions, (2) DRP resolution at 6-month follow-up, and (3) the number of additional DRPs identified by CPs beyond those detected by MDs.

**Fig. 1:**
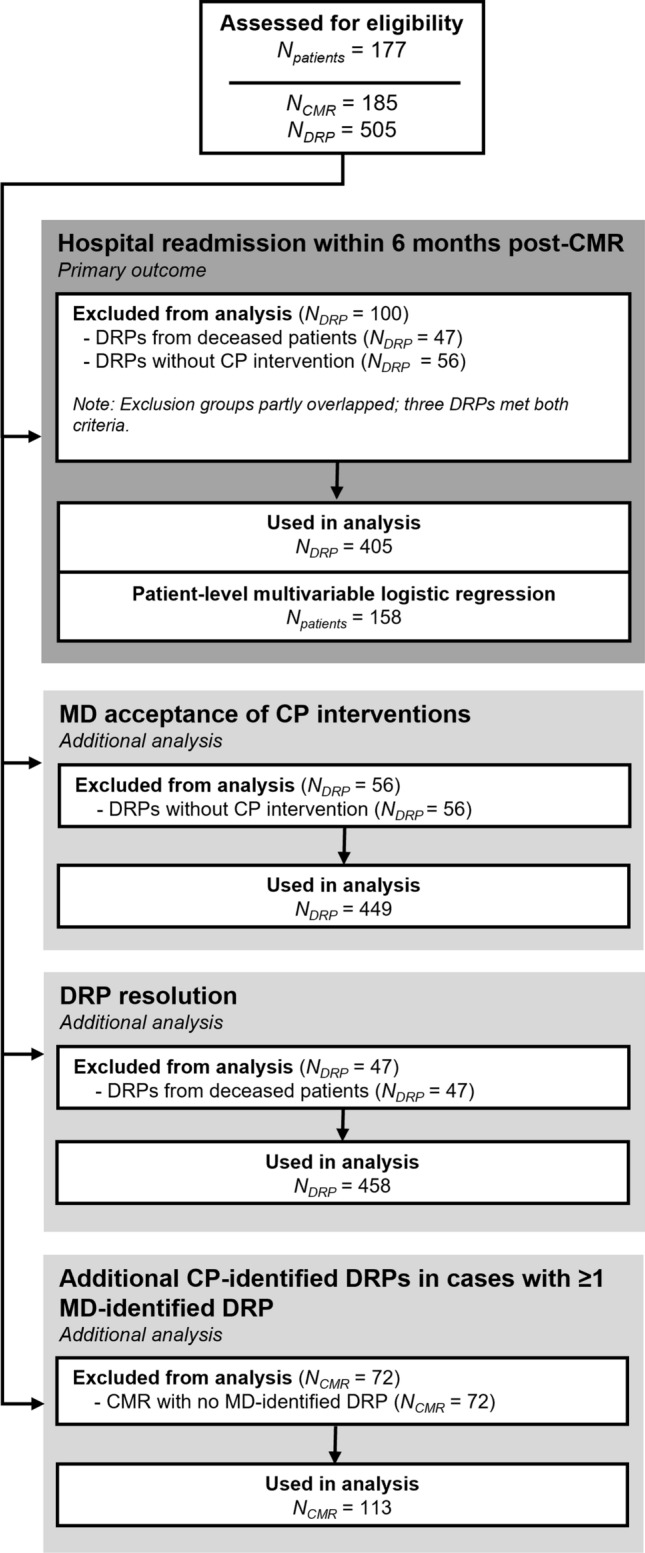
Flow diagram of DRP selection and final analytic dataset. DRP: drug-related problem; CMR: comprehensive medication review; CP: clinical pharmacist; MD: medical doctor

All identified DRPs were included in the descriptive DRP characterization (Fig. 1). For readmission-related analyses, patients who died during follow-up were excluded from the patient-level regression, and DRPs from deceased patients were excluded from DRP-level readmission analyses, as hospital readmission could not occur in these cases. DRPs without a CP intervention were also excluded from DRP-level readmission analyses.

For additional DRP-related analyses, inclusion criteria differed by outcome. For MD’s acceptance, only DRPs with a CP-proposed intervention were included. For DRP resolution, only DRPs from deceased patients were excluded, while DRPs without CP intervention were retained in the analysis, as resolution through other mechanisms was still possible. For the analysis of additional DRPs identified by CPs, only CMRs with ≥ 1 MD-identified DRP were included.

### Statistical analysis

Statistical analysis was performed using Jamovi (version 2.6.19). Univariate analysis was performed using Mann–Whitney test for non-normally distributed continuous data, while either Fisher’s exact test or the Chi-square test was used for categorical data. Paired data were analysed using the Wilcoxon signed-rank test. Patient-level multivariable logistic regression examined the association between 6-month hospital readmission and potential predictors: age, sex, comorbidity count, number of CMRs, medication count, total DRP count, presence of any unresolved DRPs, and the number of unresolved DRPs following the CMR. Covariates were selected based on clinical relevance and commonly reported predictors of hospital readmission and were retained in the model irrespective of statistical significance. The number of CMRs per patient (range 1–3; only seven patients had more than one review) was included as an exploratory covariate to account for potential differences in patient complexity, as patients receiving multiple reviews may represent a subgroup with higher clinical burden and baseline risk of readmission. Both the presence of any unresolved DRP (binary) and the number of unresolved DRPs (count) were included to evaluate potential threshold and dose–response associations with readmission. Continuous variables were included as such, while sex and presence of unresolved DRPs were treated as categorical. Model fit was assessed using McFadden’s R^2^, Cox & Snell R^2^, and Nagelkerke R^2^. Multicollinearity was assessed using variance inflation factors (VIF). All tests were two-tailed; *p* < 0.05 was considered statistically significant.

### Ethics approval

The study was conducted according to the guidelines of the Declaration of Helsinki and approved by The National Medical Ethics Committee of the Republic of Slovenia (Approval No. 0120–159/2022/7; dated 26 May 2022). Informed consent was waived due to the retrospective nature of the study.

## Results

### Patient demographics and clinical characteristics

A total of 177 patients were included (median age 58 years, IQR 45–72; 51.4% male). They were taking a median of 9 medications (IQR 6–13) at the time of the CMR. Comorbidities were common: 63.3% of patients had at least one of the specified chronic conditions. Table 1 summarises the baseline demographic and clinical characteristics.

**Table 1:** Demographic and clinical characteristics of patients (*N* = 177)

Characteristic	Value
*Demographics*	
Age, years (median, IQR)	58 (45–72)
Sex	91 male (51.4%); 86 female (48.6%)
Patient setting at time of CMR	109 inpatient (61.6%); 68 outpatient (38.4%)
*Clinical data*	
No. of medications (median, IQR)	9 (6–13)
Comorbidities (No. of patients with):	
Essential (primary) hypertension	87 (49.2%)
Diabetes mellitus	39 (22.0%)
Chronic heart failure	30 (16.9%)
Chronic atrial fibrillation	28 (15.8%)
Chronic kidney disease	26 (14.7%)
Major depressive disorder	29 (16.4%)
*6-month outcomes*^a^	
Hospital readmission (any cause) (%)	35 (19.8%)
No hospital readmission (%)	123 (69.5%)
Died within 6-month follow-up (%)	19 (10.7%)

^a^Outcomes were assessed in the 6 months following the patient’s CMR

*IQR, interquartile range; CMR, comprehensive medication review

### DRP burden and characteristics

#### Comprehensive medication reviews and identified DRPs

One hundred and seventy-seven patients underwent 185 CMRs. Most patients (170) received a single CMR, while 6 patients underwent two reviews, and 1 patient underwent three reviews. A total of 505 DRPs were identified (mean 2.7 DRPs per CMR; SD = 2.0, range 1–11).

#### DRP frequency and risk factors

Patients prescribed five or more medications had more DRPs than those with fewer than five (median 3.0 vs. 1.0; *p* = 0.001). Patients with two or more comorbidities had a greater DRP burden than those with fewer than two (median 3.0 vs. 1.0; *p* < 0.001). Older patients (> 65 years) had more DRPs than those aged ≤ 65 (median 3.0 [IQR: 2–5]) vs. 2.0 [IQR: 1–3]; *p* = 0.010).

#### DRP characteristics, categories and causes by identifier

Most DRPs were classified as potential rather than clinically expressed (74.9% vs 25.1%). CPs identified the majority of all DRPs (73.9%), particularly potential DRPs, while MDs more often documented clinically expressed DRPs. Identification differed significantly between MDs and CPs (*χ*^2^ = 8.93, *p* = 0.003), with MDs less likely to identify potential DRPs than CPs (OR = 0.519; 95% CI 0.34–0.80).

Across PCNE problem categories (e.g., treatment effectiveness, treatment safety, other), CPs more frequently identified treatment effectiveness and other medication-related issues, while MDs more often identified non-specific problems (e.g., unclear problems).

The association between DRP cause and identifier was statistically significant (*χ*^2^ = 57.6, *p* < 0.001), with CPs predominantly identifying DRPs with clearly defined pharmacotherapeutic causes (e.g., drug and dose selection). Detailed distribution of DRP types, categories, causes and corresponding intervention acceptance and resolution rates are presented in Table 2.

**Table 2:** Interventions proposed by CPs, acceptance rates by MDs and resolution rates by DRP type, category and cause

Measure or group	Number of DRPs (*n*, % of total)	DRP identification			Intervention acceptance rate (%)	*p*-value	DRP resolution rate (%)	*p*-value
		CP (n, % of category)	MD (*n*, % of category)	*p*-value				
Total DRPs	505 (100)	373 (73.9)	132 (26.1)	N/A	N/A	N/A	59.2	N/A
DRPs with CP intervention	449 (88.9)	348 (77.5)	101 (22.5)	N/A	70.2	N/A	89.8^**a**^	N/A
*DRP by type*								
Potential DRPs	378 (74.9)	292 (77.2)	86 (22.8)	**0.003**	68.6	0.220	60.3	0.375
Clinically expressed DRPs	127 (25.1)	81 (63.8)	46 (36.2)	N/A	74.8	N/A	55.7	N/A
*DRP by category*								
**Treatment effectiveness**	137 (27.1)	112 (81.8)	25 (18.2)	**0.014**	65.9	0.210	63.6	0.758
No effect of drug treatment despite correct use	4 (0.8)	3 (75.0)	1 (25.0)	1.000†	100.0	0.322†	100.0	0.303†
Effect of drug treatment not optimal	117 (23.2)	94 (80.3)	23 (19.7)	0.069	63.6	0.085	61.8	0.383
Untreated symptoms or indication	16 (3.2)	15 (93.8)	1 (6.2)	0.082†	73.4	0.784	73.3	0.504
**Treatment safety** (ADE (possibly) occurring)	258 (51.1)	192 (74.4)	66 (25.6)	0.771	69.0	0.569	57.7	0.533
**Other**	110 (21.8)	72 (65.5)	38 (34.5)	**0.023**	73.7	0.397	56.6	0.143
Unnecessary drug-treatment	65 (12.9)	61 (93.8)	4 (6.2)	** < 0.001**†	75.0	0.378	67.2	0.143
Unclear problem/complaint	45 (8.9)	10 (22.2)	35 (77.8)	** < 0.001**†	75.6	0.423	65.8	0.386
*DRP by cause*								
Drug selection	275 (54.4)	216 (78.5)	59 (21.5)	**0.009**	69.1	0.577	58.6	0.799
Drug form	5 (1.0)	4 (80.0)	1 (20.0)	1.000†	80.0	1.000†	80.0	0.653†
Dose selection	69 (13.7)	58 (84.1)	11 (15.9)	**0.038**	70.6	0.933	65.0	0.324
Treatment duration	10 (2.0)	10 (100.0)	0 (0.0)	0.070†	70.0	1.000†	70.0	0.538†
Dispensing	1 (0.2)	1 (100.0)	0 (0.0)	1.000†	100.0	1.000†	100.0	1.000†
Drug use process	12 (2.4)	12 (100.0)	0 (0.0)	**0.042†**	75.0	1.000†	66.7	1.000†
Patient-related	12 (2.4)	12 (100.0)	0 (0.0)	**0.042†**	41.7	1.000†	50.0	1.000†
Patient-transfer related	1 (0.2)	1 (100.0)	0 (0.0)	1.000†	100.0	1.000†	100.0	1.000†
Other (including TDM)	120 (23.7)	59 (49.2)	61 (50.8)	** < 0.001**	74.7	0.286	54.6	0.272

Denominators for percentages vary by analysis. Denominators used for percentages calculations:

**Identification (*n* = 505):** rates are based on all identified DRPs. **Intervention acceptance (*n* = 449):** rates are based on DRPs with a CP-proposed intervention. **Resolution (*n* = 458):** rates are based on evaluable DRPs (excluding DRPs from patients who died during follow-up).

^a^Resolution among DRPs with accepted interventions: 283/315 (89.8%).

*p*-values reflect comparisons of identification, intervention acceptance and resolution rates across DRP subgroups using the Chi-squared test; †Fisher’s exact test used due to small expected counts. Bold values indicate statistical significance (*p* < 0.05).

While CPs documented most DRPs overall, the extent to which this represents additional clinical value beyond MD-identified DRPs is addressed in the subsequent section on pharmacist contribution.

### DRP-related analysis (pharmacists’ contribution)

#### Additional DRPs identified beyond those detected by MDs

In 113 CMRs with ≥ 1 MD-identified DRP, CPs identified additional DRPs. These reviews involved 110 patients, with three patients undergoing two CMRs. On average, CPs found 1.21 additional DRPs per CMR (median 1, SD 1.64), demonstrating additional yield of DRPs identified. A one-sample Wilcoxon signed-rank test showed a significant difference from zero (*p* < 0.001).

#### Interventions proposed by CPs and MDs’ acceptance

CPs proposed at least one intervention for 88.9% of DRPs. MDs accepted 70.2% of recommendations (95% CI: 65.7–74.4%). Acceptance did not differ by DRP type, category, or cause (Table 2).

#### DRP resolution

At follow-up, 271/458 DRPs were fully or partially resolved (59.2%, 95% CI: 54.5–63.7%), while 187 remained unresolved (47 DRPs were not evaluable due to patient death). Resolution was not associated with DRP type, category or cause. Rates were slightly higher for potential versus clinically expressed DRPs (60.3% vs 55.7%, *p* = 0.375) and did not differ across categories (*χ*^2^ = 1.57, *p* = 0.456) or causes (*χ*^2^ = 5.26, *p* = 0.730). Details are shown in Table 2.

### Primary outcome: association between DRP resolution and hospital readmission

**Fig. 2:**
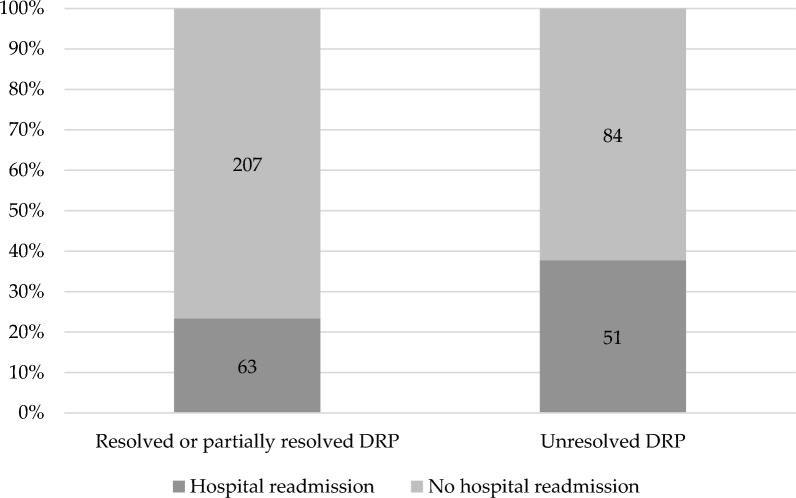
Association between DRP Resolution and Hospital Readmission Within 6 Months of CMR

We examined the relationship between DRP resolution status and hospital readmission 6 months post-CMR. In a DRP-level comparison of readmission outcomes, patients were readmitted in 23.3% of cases where a DRP was resolved or partially resolved, compared to 37.8% of cases where a DRP remained unresolved (*χ*^2^ = 9.28, *p* = 0.002). Resolved or partially resolved DRPs were associated with lower odds of readmission within 6 months for the corresponding patient (OR = 0.50, 95% CI: 0.32–0.78) and a lower relative risk of readmission (RR = 0.62). Because patients could contribute multiple DRPs, this DRP-level comparison does not account for within-patient clustering and is presented for descriptive purposes. The relationship between DRP resolution and hospital readmission is illustrated in Fig. 2.

There was an association between MDs’ acceptance of CP interventions and DRP resolution (*χ*^2^ = 281, *p* < 0.001). Higher DRP resolution rates were observed when interventions were accepted (89.8% vs. 7.5%; RR = 4.71, 95% CI: 3.45–6.42). Acceptance of CP interventions was also associated with lower readmission rates in a DRP-level comparison of readmission outcomes (24.5% vs. 37.4%; χ^2^ = 6.78, *p* = 0.009; OR = 0.54, RR = 0.66).

### Multivariable analysis of readmission predictors

To account for clustering of multiple DRPs within patients, a patient-level logistic regression (Table 3) was conducted to identify factors independently associated with hospital readmission 6 months post-CMR (N = 158 patients). The model included age, sex, number of CMRs, comorbidity count, medication count, total DRP count, number of unresolved DRPs, and the presence of any unresolved DRPs after CMR. The presence of any unresolved DRPs demonstrated the largest effect, with patients having unresolved DRPs showing higher odds of readmission compared to those without unresolved DRPs (OR = 4.02, 95% CI: 1.12–14.39, *p* = 0.033). In addition, a higher number of unresolved DRPs was independently associated with increased readmission (OR = 1.74, 95% CI: 1.10–2.74, *p* = 0.017). A higher number of comorbidities was also associated with higher readmission (OR = 1.47, 95% CI: 1.01–2.15, *p* = 0.046). Age, sex, the number of CMRs, medication count, and total DRP count were not significant predictors (all *p* > 0.05). The model demonstrated acceptable explanatory power (McFadden’s R^2^ = 0.116, Nagelkerke R^2^ = 0.177). No evidence of multicollinearity was observed (all VIF < 4).

**Table 3:** Patient-level logistic regression model predicting hospital readmission within 6 months following CMR (N = 158)

Predictor	Odds ratio	95% CI	*p*-value
Age (per year)	0.98	0.95–1.01	0.126
Sex (female vs. male)	1.14	0.50–2.57	0.757
Number of CMRs per patient (per CMR)	2.27	0.60–8.64	0.230
Comorbidity count (per condition)	1.47	1.01–2.15	**0.046**
Medication count (per medication)	1.08	0.97–1.20	0.144
Total DRP count (per DRP)	0.76	0.53–1.09	0.129
Number of unresolved DRPs (per DRP)	1.74	1.10–2.74	**0.017**
Presence of unresolved DRPs (yes vs. no)	4.02	1.12–14.39	**0.033**

Bolded values indicate statistical significance (*p* < 0.05). Odds ratios represent the odds of hospital readmission. Model fit: McFadden’s R^2^ = 0.116, Cox & Snell R^2^ = 0.116, Nagelkerke R^2^ = 0.177, Tjur R^2^ = 0.131. Patients who died during follow-up (*n* = 19) were excluded from the analysis. Only seven patients underwent more than one CMR; the estimate for the number of CMRs per patient should be interpreted cautiously.

## Discussion

In this retrospective study, a patient-level analysis revealed that the presence of any unresolved DRP was independently associated with a four-fold increase in the odds of 6-month hospital readmission (OR = 4.02). A dose–response pattern was observed, with each additional unresolved DRP increasing readmission odds by 74% (OR = 1.74 per unresolved DRP). Comorbidity burden was also independently associated with readmission (OR = 1.47), indicating higher patient complexity. In contrast, the total number of DRPs identified was not associated with readmission, suggesting that the persistence of DRPs, rather than their detection, may be clinically relevant.

In a complementary DRP-level comparison of readmission outcomes (not accounting for within patient clustering), patients were readmitted in 23.3% of instances where a DRP was resolved or partially resolved, compared with 37.8% where a DRP remained unresolved (*p* = 0.002). Lower readmission rates were observed when MDs accepted CPs’ recommendations (24.5% vs. 37.4%, *p* = 0.009). Non-accepted recommendations were more often associated with unresolved DRPs and higher readmission rates. The physician acceptance rate (70.2%) was consistent with previous reports (70–90%) [11–17] and systematic review estimates (39% to 100%) [19].

Our findings highlight the potential clinical relevance of unresolved DRPs; however, given the observational design, causal conclusions regarding the effectiveness of pharmacist-led CMRs compared with usual care cannot be drawn. Unresolved DRPs were associated with a higher probability of readmission. Consideration of the nature of unresolved DRPs may help interpret the association with readmission. Unresolved DRPs likely represent medication-related problems that persist despite identification and may reflect issues requiring optimisation and monitoring (e.g., persistent suboptimal therapy or ongoing safety concerns), while also serving as markers of higher patient complexity, illness severity and residual risk independent of the DRP itself. They may help identify patients who could benefit from prioritised follow-up and medication optimisation within pharmacist-physician collaboration.

Strengths include the systematic use of the PCNE classification and a diverse patient population with complete follow-up data. Unlike many prior studies limited to single wards or specific patient groups, this study spanned a broad range of settings. By evaluating a clinical outcome (readmissions) alongside key DRP-related process measures (intervention acceptance, DRP resolution, and the added DRP yield of pharmacists) it provides a comprehensive understanding of the pharmacist’s role.

Limitations include retrospective design and potential selection bias, as all patients were referred by MDs for a CMR (likely representing a higher-risk population), limiting generalizability. Residual confounding cannot be excluded, as factors such as disease severity, social determinants of health, and treatment adherence were not available and may have influenced both DRP resolution and hospital readmission. Lack of a non-CMR comparison group prevents evaluation of CPs intervention effectiveness compared with usual care. Excluding patients who died during follow-up may underestimate associations, as mortality is a competing outcome for hospital readmission. If unresolved DRPs are also associated with mortality, patients with the highest burden may die before experiencing readmission, resulting in a conservative estimate of the association.

At the time of data collection, hospital information systems in Slovenia were not nationally integrated. Readmissions to hospitals other than the study centre could not be captured, possibly leading to underestimation or misclassification. Only seven patients underwent more than one CMR. For these patients, only the most recent review was linked to readmission, which may not capture cumulative effects of earlier reviews. Given that only seven patients underwent more than one CMR, the variable “number of CMRs per patient” in the logistic regression had limited variability, restricting both its interpretability and statistical power, and raising the possibility of reverse causality, where patients at higher risk of readmission may have been more likely to receive multiple reviews.

The primary analysis was conducted at the patient level to avoid within-patient clustering. DRP-level comparisons of readmission outcomes remain subject to within-patient correlation. The modest number of outcome events relative to predictors may limit model stability and widen confidence intervals.

As a single-centre study conducted in a setting with an established clinical pharmacy service, the external validity of the findings to other contexts may be limited. These limitations should be considered when interpreting the observed association between unresolved DRPs and hospital readmission.

Not only identification, but also clinical uptake of CP recommendations matter. The high physician acceptance rate reflects strong interprofessional collaboration and reinforces the value of CP contributions. Where MDs themselves had identified a DRP, CPs consistently detected additional issues. Our findings support the potential benefit of a team-based approach to medication management, where CPs’ clinical input is both valued and acted upon. Prospective studies, ideally randomized, are needed to determine whether interventions that successfully resolve DRPs can causally reduce hospital readmissions. Studies should also include broader populations, not just high-risk patients.

## Conclusion

In this retrospective study, unresolved DRPs were associated with a higher risk of hospital readmission within 6 months. These findings highlight unresolved DRPs as a strong, independent marker of increased readmission risk. Integrating pharmacists within multidisciplinary teams appears justified, although larger prospective studies are needed to determine whether interventions targeting the resolution of these problems can causally reduce readmissions.

## Data Availability

The datasets generated and analysed during the study are available from the corresponding author on reasonable request.
